# Clinicopathological‐genetic features of congenital myasthenic syndrome from a Chinese neuromuscular centre

**DOI:** 10.1111/jcmm.17417

**Published:** 2022-06-06

**Authors:** Kun Huang, Hui‐Qian Duan, Qiu‐Xiang Li, Yue‐Bei Luo, Fang‐Fang Bi, Huan Yang

**Affiliations:** ^1^ Department of Neurology, Xiangya Hospital Central South University Changsha China; ^2^ National Clinical Research Center for Geriatric Disorders, Xiangya Hospital Central South University Changsha China; ^3^ Institute of Molecular Precision Medicine and Hunan Key Laboratory of Molecular Precision Medicine, Xiangya Hospital Central South University Changsha China

**Keywords:** congenital myasthenic syndrome, myopathy, neuromuscular disorder, pathology

## Abstract

Congenital myasthenic syndrome (CMS) encompasses a heterogeneous group of inherited disorders affecting nerve transmission across the neuromuscular junction. The aim of this study was to characterize the clinical, physiological, pathohistological and genetic features of nine unrelated Chinese patients with CMS from a single neuromuscular centre. A total of nine patients aged from neonates to 34 years were enrolled who exhibited initial symptoms. Physical examinations revealed that all patients exhibited muscle weakness. Muscle biopsies demonstrated multiple myopathological changes, including increased fibre size variation, myofibrillar network disarray, necrosis, myofiber grouping, regeneration, fibre atrophy and angular fibres. Genetic testing revealed six different mutated genes, including AGRN (2/9), CHRNE (1/9), GFPT1 (1/9), GMPPB (1/9), PLEC (3/9) and SCN4A (1/9). In addition, patients exhibited differential responses to pharmacological treatment. Prompt utilization of genetic testing will identify novel variants and expand our understanding of the phenotype of this rare syndrome. Our findings contribute to the clinical, pathohistological and genetic spectrum of congenital myasthenic syndrome in China.

## 1 INTRODUCTION

Congenital myasthenic syndrome (CMS) encompasses a heterogeneous group of inherited disorders affecting nerve transmission across the neuromuscular junction.^1^ The incidence of CMS was estimated to be 1.8–22.2 per million; however, due to the complexity of the procedures that are used to obtain an accurate diagnosis, incidence rates are likely underestimated.^2^, ^3^, ^4^, ^5^, ^6^ Currently, more than 30 proteins are known to be involved in various types of CMS. Generally, proteins related to CMS are located at the presynaptic, synaptic or postsynaptic region of the neuromuscular junction (NMJ), or they undergo abnormal glycosylation. Among them, mutations in *CHRNE*, *RAPSN* and *COLQ* are the most frequent, accounting for half of CMS cases in a large‐scale analysis including 680 patients.^7^ Mutations in CMS‐related genes can be used to accurately diagnose CMS.

Generally, CMS is characterized by fatigability or skeletal muscle weakness with an onset at birth to early childhood. Electromyographic findings of a decrease in repetitive nerve stimulation (RNS) and increased jitter on single‐fibre electromyography (EMG) may support the diagnosis.^8^ Genetic testing or whole‐exome sequencing could establish a diagnosis of CMS and guide pharmacological treatment. For example, β2‐receptor agonist therapy could be the first‐choice pharmacological strategy for treating CMS with *DOK7* and could be a supplementary treatment for CMS with *CHRNE* mutations.^9^, ^10^ Specifically, AChE inhibitors should also be avoided in patients with CMS due to AChE deficiency, such as CMS with *COLQ* mutations or DOK7 mutations.^10^, ^11^, ^12^

There is an ever‐expanding panel of mutations associated with CMS. There are several case reports of Chinese CMS patients, and one study described 35 CMS patients from the northern part of China.^13^ In this study, we describe nine patients from the southern part of China diagnosed with CMS to expand the clinical and pathological spectrum of CMS.

## 2 MATERIALS AND METHODS

### 2.1 Ethics approval and patients

With the approval of the Ethics Committee of Xiangya Hospital, Central South University, a total of nine Chinese patients in the Neuromuscular Center of Xiangya Hospital of Central South University from 2015 to 2020 were recruited, as mentioned in previous research.^14^, ^15^ The diagnosis of CMS was independently established by at least two neurologists according to clinical manifestations, pathological changes, EMG and genetic mutations.

### 2.2 Serum antibody test

Serum samples were routinely tested by the DAAN Clinical Laboratory Central (Guangzhou, China) using an enzyme‐linked immunosorbent assay (ELISA) method, and titres >0.45 nmol/L for anti‐AChR antibody and >9.5 pmol/L for anti‐MuSK antibody were defined as positive.

### 2.3 Electromyography

All repetitive nerve stimulation (RNS) tests were performed at rest and with low‐frequency (3 Hz) and high‐frequency (20 Hz) stimulation in the following nerves: facial nerve, accessory nerve, peroneal nerve, spinal accessory nerve, nervus peroneus communis, median nerve, nervus tibialis, sural nerve and ulnar nerve. A decremental response in amplitude at the fourth potential that was higher than 10% compared with the first potential was considered abnormal.^16^ Other EMG signals, such as spontaneous muscle activity or compound muscle action potential (CMAP), were also recorded.

### 2.4 Biopsies and pathological examination

Open muscle biopsies were taken from the left biceps brachii muscles in all patients, except for patient 1, whose open muscle biopsy was taken from the left gastrocnemius muscles. The muscle tissue was immediately frozen in isopentane cooled with liquid nitrogen and stored at −80°C. Immunohistochemical staining was performed as described elsewhere with minor modifications.^17^, ^18^, ^19^, ^20^ Briefly, histological and immunohistochemical analyses were performed on 5 μm thick sections using a cryostat. Routine histological staining of muscle sections was performed using haematoxylin and eosin (HE), modified Gömöri trichrome, acid phosphatase, periodic acid‐Schiff, oil red O (ORO), nicotinamide adenine dinucleotide dehydrogenase‐tetrazolium reductase (NADHTR), adenosine triphosphatase (ATPase) (pH 4.3, 4.6, 11.0), succinic dehydrogenase (SDH) and cytochrome C oxidase.

### 2.5 Genetic testing

Genetic testing was performed as we previously reported with minor modifications.^21^ Briefly, genomic DNA (gDNA) was extracted using a DNeasy Blood & Tissue Kit (Qiagen) according to the manufacturer's instructions. gDNA samples were sent to GUANGZHOU JIAJIAN MEDICAL TESTING, and next‐generation sequencing (NGS) analysis, which targeted the exons and exon–intron junctions of 1546 genes known to be associated with hereditary neuropathies and myopathies, was conducted. The sequences obtained were compared with those in the human genome database. Samples were sequenced on a HiSeq X Ten (Illumina) using 2 × 150 paired‐end sequencing. The genetic study sequenced the reference genome to 30× average depth and achieved 95% coverage. Sequences were aligned to the reference human genome (hg19) sequence using the Burrows–Wheeler Alignment tool (BWA 0.7.12) with default parameters. Detected sequence variants, if present in the dbSNP, HapMap, 1000 Genome, ESP6500, ExAC or in‐house Chinese Exome Database (1500 Chinese Han individuals), were all removed. Sanger sequencing was performed in the patients' parents to confirm heredity.

## 3 RESULTS

### 3.1 Demographic and clinical features

In our neuromuscular centre, we retrospectively screened patients diagnosed with CMS from 2015 to 2020 and found nine patients with CMS who were enrolled. All patients were Han Chinese and were from the southern part of China. All patients were male, and the median age of onset, disease duration and follow‐up duration were 8 years (interquartile range, 5–18), 7 years (interquartile range, 3–10) and 12 months (interquartile range, 9–13), respectively (Table 1).

**TABLE 1:** Clinical features and physical examination of the nine patients with CMS

Case	Gender	Onset age (years)	Disease duration (years)	Symptoms	Tendon reflex	Gowers sign	Follow up duration (months)
1	M	18	7	Muscle weakness of proximal and mainly distal of all limbs, facial muscle weakness, ptosis	Normal	+	12
2	M	8	8	Muscle weakness of proximal and distal of all limbs, facial muscle weakness, ptosis	Normal	+	18
3	M	Neonate	20	Muscle weakness of proximal and distal of all limbs, ptosis, dysphagia	Normal	−	6
4	M	7	10	Muscle weakness of proximal limbs	Normal	−	9
5	M	34	2	Muscle weakness of proximal and distal of all limbs, ptosis, dysphagia	Decreased	+	13
6	M	5	15	Muscle weakness of proximal and distal of lower limbs, facial muscle weakness, ptosis	Absent	+	12
7	M	8	1	Muscle weakness of proximal and distal of upper limbs, facial muscle weakness, ptosis	Normal	−	14
8	M	1	6	Muscle weakness of proximal and distal of all limbs, ptosis, tachycardia	Absent	+	8
9	M	25	3	Muscle weakness and atrophy of distal lower limbs, facial muscle weakness, ptosis	Decreased	+	10

All the patients denied having any family history of muscular disorders. All patients were ambulatory. All patients presented with varying degrees of limb weakness. Eight patients exhibited ptosis, five patients had facial muscle weakness, two patients presented with dysphagia and one patient had tachycardia. Two patients had an absent tendon reflex, and two had a decreased tendon reflex. Six patients were positive for the Gowers sign. More details regarding the demographic and clinical features are shown in Table 1.

Except for patient 8, who lacked serum creatine kinase, the levels of creatine kinase were normal (20–170 U/L) in four patients, while the other four patients had low to moderately increased creatine kinase (Table 2). Specifically, one patient with *GMPPB* mutations had the highest creatine kinase at 2696 U/L (Table 2). All patients were AChR antibody‐negative. In addition, patients 1, 4 and 7 were also tested for MuSK antibody titres, and all three were seronegative for MuSK antibody.

**TABLE 2:** Examinations and treatments of the nine patients with CMS

Case	Mutated gene	Age of examination	CK level (U/L)	EMG	Muscle biopsy	Treatment (Drug/response)
1	*AGRN*	25	142	Myopathic changes (abductor hallucis), decremental response to RNS (facial nerve, accessory nerve)	IFSV, sporadic necrotic fibre and angular fibre, myofibrillar network disarray, small group atrophy, type I myofiber grouping	Slight improvement to pyridostigmine
2	*AGRN*	16	598	Myopathic changes (medial vastus muscle)	IFSV, sporadic necrotic fibre and regenerative fibre, myofibrillar network disarray, type II myofiber grouping	Deterioration with pyridostigmine, moderate improvement to albuterol
3	*CHRNE*	20	143	Decremental response to RNS (facial nerve, accessory nerve)	IFSV, hyperplasia of connective tissue	Great improvement to pyridostigmine; no response to prednisone
4	*GFPT1*	17	307	Myopathic changes (biceps brachii, triceps brachii), decremental response to RNS (nervus peroneus communis)	IFSV, angular myofiber, tubular aggregates, myofibrillar network disarray	Great improvement to pyridostigmine
5	*GMPPB*	36	2696	Myopathic changes (biceps brachii, abductor hallucis), decremental response to RNS (ulnar nerve)	IFSV, sporadic necrotic fibre and regenerative fibre, CN, myofibrillar network disarray, moth‐eaten fibre	Great improvement to pyridostigmine; no response to prednisone+ azathioprine
6	*PLEC*	20	163	Myopathic changes (orbicular muscle of mouth, tibialis anterior), decremental response to RNS (facial nerve, median nerve)	IFSV, CN, fibre splitting, myofibrillar network disarray, type II myofiber grouping	Moderate improvement to pyridostigmine; no response to prednisone
7	*PLEC*	9	131	Myopathic changes (biceps brachii, triceps brachii), decremental response to RNS (ulnar nerve)	IFSV, sporadic atrophic fibre, sporadic inflammatory infiltration	No response to pyridostigmine, moderate improvement to 3,4‐DAP
8	*PLEC*	7	N/A	Normal	Slight IFSV, myofibrillar network disarray, possible tubular aggregates	Moderate improvement to pyridostigmine; no response to prednisone+ azathioprine
9	*SCN4A*	28	209	Myopathic changes (tibialis anterior), decremental response to RNS (sural nerve)	IFSV, CN, sporadic atrophic fibre, moth‐eaten fibre, type II myofiber grouping	Slight improvement to pyridostigmine

Abbreviations: CK, creatine kinase; CN, centralized nuclei; IFSV, increased fibre size variation; N/A, not available; RNS, repetitive nerve stimulation.

### 3.2 Electromyography

Electromyography revealed myopathic changes in seven patients (7/9) and a decremental response to RNS (7/9) (Table 2). Except for patient 9 (an SCN4A‐CMS patient), who showed a decremental response to high‐frequency (20 Hz) RNS, all the other patients showed a decremental response to low‐frequency (3 Hz) RNS. One patient (patient 8) with a *PLEC* mutation exhibited normal EMG. Due to technical limitations, single fibre electromyography (SFEMG) was not available at our centre.

### 3.3 Muscle pathology

**FIGURE 1:**
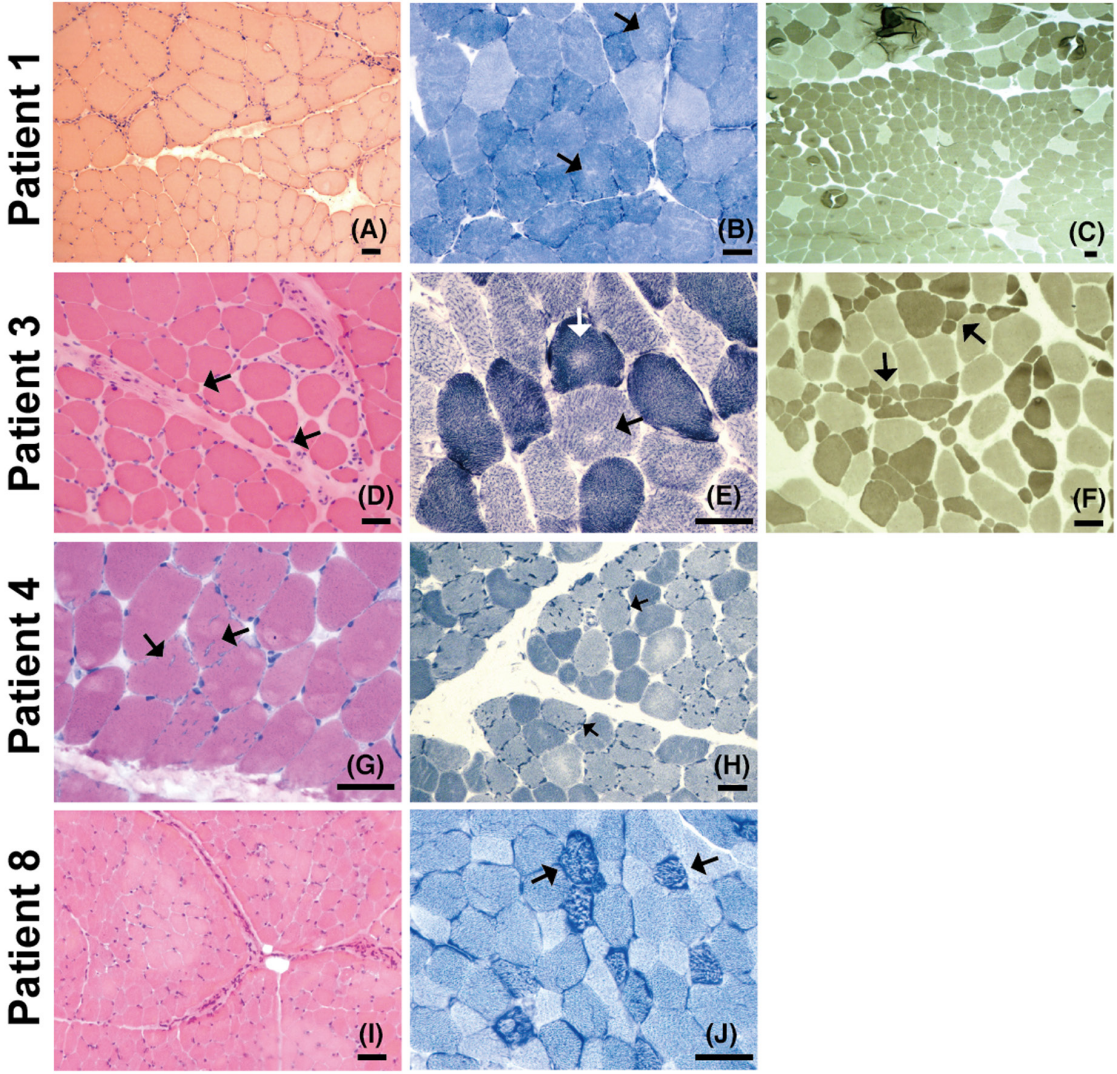
Myopathological changes in CMS patients. Patient 1’s biopsy was taken from the left gastrocnemius muscles, and the others were taken from the left biceps brachii muscles. (A) HE staining showing increased fibre size variation and a group of severely atrophic angulated fibres. (B) NADH staining showing multiple and tiny areas of uneven oxidative staining and increased subsarcolemmal activity. (C) ATPase staining (pH 11.0) showing one fascicle composed of type 2 fibres with apparently reduced diameter. (D) HE staining showing increased fibre size variation with perifascicular fibre atrophy and increased endomysial fibrosis. (E) NADH staining showing uneven areas of oxidative reaction in both type 1 and type 2 fibres. (F) APTase staining (pH 11.0) showing type 2 fibre atrophy. (G) HE staining showing the presence of multiple vacuoles with a rim of basophilic material. (H) NADH staining showing multiple hyperintense dotty areas in type 2 fibres corresponding to tubular aggregates. (I) HE staining showing the slightly increased fibre size variation. (J) NADH staining showing multiple hyperintense dotty areas possibly corresponding to tubular aggregates. Scale bar = 50 μm

All patients underwent open skeletal muscle biopsies. In the muscle biopsy specimens, we detected slightly to mildly increased fibre size variations in all patients (9/9), myofibrillar network disarray in five patients (5/9), myofiber grouping in four patients (4/9), necrosis in three patients (3/9), fibre atrophy in three patients (3/9), regeneration in two patients (2/9) and angular fibres in two patients (2/9) (Table 2, Figure 1).

### 3.4 Genetic testing and mutations

Mutation analysis of 1546 genes known to be associated with hereditary neuropathies and myopathies was performed for all patients. The following mutations associated with CMS were confirmed: *AGRN* (2/9), *CHRNE* (1/9), *GFPT1* (1/9), *GMPPB* (1/9), *PLEC* (3/9) and *SCN4A* (1/9) (Table 3). All patients exhibited autosomal recessive inheritance (either homozygous or compound heterozygous). All parents of the patients carried the genetic variants in trans. In patients with variants of unknown significance (VUS), those variants were determined to be likely damaging or deleterious by Polyphen‐2, SIFT or Mutation Taster predictive mutation impact software models in all patients, except for patient 3, whose *CHRNE* mutation was a nonsense mutation, and patient 5, whose *GFPT1* mutations were reported as pathologic.^22^, ^23^, ^24^

**TABLE 3:** Molecular genetic findings in nine patients with CMS

Case	Mutated gene	Transcript	Genotype	Allele frequency^a^	Zygosity	Pathogenicity prediction (Polyphen‐2/SIFT/Mutation Taster)
1	*AGRN*	NM_198576.3	c.1343G > A(p.R448Q) c.2563G > A(p.V855M)	0.00008998 0.0002622	Compound heterozygous	Benign/deleterious/disease causing Probably damaging/damaging/disease causing
2	*AGRN*	NM_198576.3	c.4735G > A(p.G1579S) c.4999G > A(p.V1667M)	0.00006654 0.00002863	Compound heterozygous	Benign/deleterious/disease causing Probably damaging/neutral/disease causing
3	*CHRNE*	NM_000080	c.414G > A(p.W138*)	NA	Homozygous	NA/NA/disease causing
4	*GFPT1*	NM_001244710	c.331C > T(p.R111C) c.635G > A(p.R212Q)	0.0001392 0.000003982	Compound heterozygous	Reported Reported
5	*GMPPB*	NM_013334	c.1151G > A(p.R384H) c.1067_1069del(p.M356_T357del)	0.00004029 NA	Compound heterozygous	Benign/deleterious/disease causing NA/NA/disease causing
6	*PLEC*	NM_201380	c.6172C > T(p.R2058W) c.4186G > A(p.E1396K)	0.0003820 0.00003993	Compound heterozygous	Probably damaging/deleterious/disease causing Benign/deleterious/disease causing
7	*PLEC*	NM_201380	c.4756C > T(p.R1586C) c.5432G > A(p.R1811Q)	0.00002263 0.0001123	Compound heterozygous	Probably damaging/neutral/disease causing Probably damaging/neutral/polymorphism
8	*PLEC*	NM_201380	c.4892A > C(p.E1631A) c.6172C > T(p.R2058W)	NA 0.0003820	Compound heterozygous	Probably damaging/neutral/disease causing Probably damaging/deleterious/disease causing
9	*SCN4A*	NM_000334	c.4252A > G(p.I1418V)	NA	Homozygous	Probably damaging/neutral/disease causing

Abbreviation: NA, not available.

^a^ Frequency in total population from gnomAD database (http://gnomad‐sg.org/).

### 3.5 Response to treatment

Pyridostigmine was the most commonly used medication. All patients were prescribed pyridostigmine, and two patients (2/9), including patient 1 and patient 9, showed a slight benefit. Two patients (patients 6 and 8) had moderate improvement in response to pyridostigmine, and three patients (patients 3, 4 and 5) displayed strong improvement with pyridostigmine. Patient 7, with *PLEC* mutations, did not respond to pyridostigmine but showed moderate improvement in response to 3,4‐diaminopyridine (3,4‐DAP). Patient 2, with *AGRN* mutations, worsened after treatment with pyridostigmine, which was replaced with albuterol with moderate improvement. Since some of the patients were misdiagnosed with MG, immunosuppressive treatments were prescribed in patients 3 and 6 (prednisone) and patients 5 and 8 (prednisone+ azathioprine). However, none of these patients benefited from immunosuppressive treatments.

## 4 DISCUSSION

In the current study, we report the clinical and genetic findings of nine patients from the southern part of China with CMS. We identified these patients with diverse genotypes and resultant variable clinical features and responses to therapy. In a large CMS cohort of primarily European origin, postsynaptic acetylcholine receptor (AChR) mutations were the most common, accounting for nearly half of the cases.^25^, ^26^ Fifty‐six percent of patients (5/9) in the current study exhibited mutations in genes that cause defects in postsynaptic AChR (one *CHRNE*, three *PLEC* and one *SCN4A*), consistent with previous research.^7^, ^13^ In addition, we reported patients with synaptic mutations and prepostsynaptic mutations, such as *AGRN*, *GFPT1* and *GMPPB*.

Missense mutations in *AGRN* cause CMS. Agrin is secreted into the synaptic basal lamina by the nerve terminal. The agrin‐Lrp4‐MuSK signalling pathway is the primary mechanism for the formation of NMJs.^27^ *AGRN* mutations in our study were located in the Kazal‐like 4 and Laminin EGF‐like 2 domains (patient 1) and EGF‐like 2 and Laminin G‐like 2 domains (patient 2). Kazal‐like domains function as protease inhibitors, contributing to the maintenance of a long‐lasting synaptic structure.^28^, ^29^ EGF‐like domains, which are not essential for AChR clustering, play an attenuating role in the authentic AGRN molecule.^30^ Laminin G‐like domains mediate the binding of AGRN to heparin and the cell surface receptor alpha‐dystroglycan, which are crucial to basement membrane assembly.^31^ Symptoms of AGRN‐CMS are versatile. Our two patients with *AGRN* mutations, consistent with other reported AGRN‐CMS Chinese patients,^32^, ^33^ did not exhibit manifestations of distal myopathy. One study, including patients from Norway and France, reported five patients with AGRN‐CMS with distal myopathy, suggesting that patients with distal limb muscle weakness should also be considered for AGRN‐CMS.^34^ Patients with CMS who have *AGRN* mutations respond quite differently to albuterol and pyridostigmine. CMS with *AGRN* mutations usually respond to albuterol but not to pyridostigmine.^35^ In our two patients, one responded to pyridostigmine, and one worsened, suggesting that pyridostigmine should be avoided in CMS patients with *AGRN* mutations.

The *CHRNE* gene encodes the epsilon subunit of AChR. Mutations in *CHRNE* fall into two groups: kinetic mutations with or without minor AChR deficiency and low‐expressor mutations with or without minor kinetic effects, which are also called primary AChR deficiency. Kinetic mutations consist of two classes: slow‐channel syndromes and fast‐channel syndromes.^36^ Patient 3 had been diagnosed with myasthenia gravis (MG) for several years and was prescribed other immunosuppressors, such as prednisolone and azathioprine. However, the treatment effect was not satisfactory, and genetic testing was conducted. After the genetic diagnosis was established, we prescribed pyridostigmine, and the patient had a good response.

GFPT1 is the rate‐limiting enzyme in the hexosamine biosynthetic pathway, which is indispensable for protein and lipid glycosylation. Mutations in *GFPT1* cause CMS characterized by fatigable muscle weakness owing to impaired neurotransmission. The precise pathomechanisms at the NMJ due to GFPT1 deficiency have yet to be discovered.^37^ In our study, the two missense mutations in the *GFPT1* mutant patient were previously reported.^22^, ^23^, ^24^ Previously reported patients with the *GFPT1* mutation p. R111C began having difficulty walking and climbing stairs in the second decade of life.^24^ The p. R111C mutation has no effect on the enzymatic activity of GFPT1.^22^ The reported patient with the p. R212Q mutation had an onset at 14 years old and presented with tubular aggregates under a light microscope.^23^ These patients with previously reported mutations all benefited from pyridostigmine and derived additional benefit from 3,4‐DAP and salbutamol. Our patient also exhibited a good response to pyridostigmine, suggesting that pyridostigmine may be the first choice for CMS patients with these specific mutations.

*GMPPB* encodes the enzyme GDP‐mannose pyrophosphorylase B, which catalyses the conversion of mannose‐1‐phosphate and GTP to GDP‐mannose,^38^ which is essential for the glycosylation of key proteins involved in neuromuscular junction development and function. Human GMPPB contains an N‐terminal pyrophosphorylase domain harbouring the conserved signature motif for nucleotide binding and transfer and a C‐terminal hexapeptide repeat domain expected to form a left‐handed beta helix structure.^39^ The compound heterozygous mutations in patient 5 were located at the C‐terminal region of the bacterial transferase hexapeptide domain. A previous study found that mutations in this region lead to abnormal folding of the GMPPB protein rather than an overall loss in protein expression, causing protein aggregates in the cytoplasm.^40^ *GMPPB* mutations impact signal transmission at the neuromuscular junction. Treatment with pyridostigmine has been reported to be effective in these patients.^40^, ^41^ Consistent with previous studies, the patient with *GMPPB* mutations in our cohort also showed great improvement after treatment with pyridostigmine.

In the muscle, PLEC reinforces the myocyte cytoarchitecture through links with the intermediate filament protein desmin and the dystrophin‐glycoprotein complex and is prominently expressed at the Z‐disk, sarcolemma and neuromuscular junction.^42^ Mutations in *PLEC* cause epidermolysis bullosa simplex (EBS), which may be associated with myopathy or myasthenia.^43^ Most cases of EBS associated with CMS are due to nonsense mutations, out‐of‐frame insertions or deletions within exon 31 or exon 32 that lead to a premature termination codon.^44^ In our study, all mutations, except for the p. E1396K mutation in the spectrin 4 repeat, were located in the central fibrous rod domain, and none of the patients showed any symptoms of EBS. Previous research found that compound heterozygous mutations in the central rod domain cause LGMD with the absence of prominent skin involvement.^45^ In previous studies, five CMS patients with *PLEC* mutations were prescribed pyridostigmine or 3,4‐DAP; four of them showed a good response to pyridostigmine and the other patient benefitted from 3,4‐DAP.^46^ In our cohort, all three patients were treated with pyridostigmine, two showed mild to moderate responses and the other patient had no response to pyridostigmine but showed moderate improvement in response to 3,4‐DAP. Combined with previously reported patients, no CMS patients with *PLEC* mutations worsened in response to pyridostigmine, suggesting that pyridostigmine may be safe and could be the first treatment considered for *PLEC* mutant CMS patients.

Skeletal muscle sodium channelopathies due to *SCN4A* gene mutations have a broad clinical spectrum.^47^ CMS is a rare phenotype of *SCN4A* mutations.^48^ Several patients with recessive mutations in *SCN4A* were diagnosed with CMS/congenital myopathy.^48^, ^49^, ^50^ Further investigations are required to understand the mechanism of fatigue in CMS/congenital myopathy caused by mutations in *SCN4A*. Since no symptoms or signs of myotonia or paralysis were found, combined with the decremental response of high (20 Hz) RNS, we established the diagnosis of CMS in this patient.

In our cohort, the most frequently observed mutations were in *AGRN* and *PLEC*. The frequency of mutant genes in our current study, which is from the southern part of China, is more similar to another cohort from the northern part of China than to frequencies from other countries. AGRN‐CMS is one of the most frequent subtypes of CMS from both the southern and northern parts of China^13^ but is ultrarare in other countries, in which CHRNE‐CMS is usually the most frequent subtype.^2^, ^4^, ^6^, ^51^ CMS caused by mutations in *GFPT1* ^52^ and *DPAGT1* ^53^ has been associated with tubular aggregates that are of diagnostic support for certain subtypes of CMS. For the therapeutic strategy, acetylcholinesterase inhibitors result in some improvement in the majority of CMS but should be avoided in some mutated CMS genes, such as *DOK7* ^10^ and *COLQ* ^54^, and should be carefully prescribed for AGRN‐CMS.^13^

The most common misdiagnosis in our cohort of CMS was MG. Seronegative MG patients unresponsive to immunosuppressive treatment should always be re‐evaluated for CMS. Another common misdiagnosis for CMS is myopathy, especially limb girdle muscular dystrophy (LGMD) since they also have elevated serum creatine kinase levels and myopathic changes in the EMG. LGMD‐CMSs are usually caused by *DOK7*, *GFPT1*, *ALG2* and *ALG14* mutations. The presence of fluctuations, daily or over longer time periods, should raise a suspicion of CMS, even in patients previously diagnosed with myopathy, including LGMD, and careful electrophysiological studies are needed, including a search for repetitive CMAP and single fibre EMG if the RNS studies are negative.^55^ Electrophysiological tests are very helpful for the differential diagnosis of CMS. Muscle biopsy usually discloses generally nonspecific findings, which play a limited role in differential diagnosis. Genetic testing should be considered for differential diagnosis. In our cohort, RNS revealed a ≥ 10% decrease in most patients (7/9). The differentiation of CMS subtypes, which is usually very difficult clinically, can be achieved using molecular genetic tests. Whole‐exome sequencing should be considered in patients suspected of having CMS.

## AUTHOR CONTRIBUTIONS

**Hui‐Qian Duan:** Data curation (equal); formal analysis (equal); investigation (equal); methodology (equal). **Qiu‐Xiang Li:** Data curation (equal); investigation (equal); methodology (equal). **Yue‐Bei Luo:** Data curation (equal); investigation (equal); methodology (equal). **Fang‐Fang Bi:** Data curation (equal); investigation (equal); methodology (equal); writing – review and editing (equal).

## CONFLICT OF INTEREST

The authors declare no conflict of interest.

## Data Availability

The original data that described in this study are available from the corresponding author upon reasonable request.
